# Human Epileptic Neurons: They Are “Sag”-nificantly Different!

**DOI:** 10.1523/ENEURO.0278-25.2025

**Published:** 2025-09-12

**Authors:** Mélina Scopin

**Affiliations:** Institut du Cerveau-Paris Brain Institute-ICM, Sorbonne Université, Inserm, CNRS, Hôpital de la Pitié Salpêtrière, Paris F-75013, France

Childhood epilepsy is a challenging and often devastating condition. A significant proportion of children experience drug-resistant seizures, which have a substantial impact on their quality of life. For these patients, surgical therapeutic removal of the epileptogenic brain tissue may be necessary. This procedure also provides a unique opportunity to characterize human epileptic neurons and the mechanisms of ictogenesis in vitro. A recent study (Kushner et al., 2025) takes advantage of postoperative tissue to investigate the role of distinct neuronal subtypes in mediating epileptic activity in the context of pediatric epilepsy.

A review of epilepsy surgeries reveals that 25% of cases are pediatric (Blumcke et al., 2017). Among these patients, 40% exhibit malformations of cortical development (MCD), including focal cortical dysplasia, hemimegaloencephaly, and tuberous sclerosis complex.

In recent years, studies in both rodents and humans have examined the specific roles of different cell types and cortical layers in the onset, propagation, and persistence of seizures. Using pharmacologically induced seizure-like activity, often with the convulsant drug 4-aminopyridine (4AP), Layer 2/3 pyramidal neurons (L2/3 PNs) were found to be rapidly and synchronously recruited at seizure initiation in rodents (Aeed et al., 2020). However, a more recent study conducted in epileptic patients using intracranial multielectrode array recordings reported contradictory results (Bourdillon et al., 2024). Current-source density analysis revealed the recruitment of deeper layer neurons at the seizure onset and the later recruitment of L2/3 during seizure propagation. The precise involvement of L2/3 neurons in seizure onset may depend on the type of epilepsy and the experimental model used. This underscores the importance of systematically analyzing their characteristics in the context of different forms of epilepsy.

The characteristics of L2/3 PNs in the human brain have been studied in the pseudocontrol cortex, i.e., the postoperative tissue sampled from regions outside the epileptic focus (Deitcher et al., 2017). The study revealed two subtypes of L2/3 PNs: profuse-tufted and slim-tufted pyramidal neurons. These neurons exhibited both functional and morphological differences. Profuse-tufted neurons displayed a higher density of tuft branches and higher firing rates. While these precise subtypes did not correspond to previously identified classes of neurons in the rodent cortex, their active biophysical properties were typical of cortical pyramidal cells in rodents. One notable exception was the enhanced function of human L2/3 PN HCN channels, which are responsible for generating a sag current upon hyperpolarization. This current may enable human L2/3 PNs to perform more complex dendritic computations and may promote rhythmic activity in a pacemaker-like manner. This analysis suggests L2/3 PNs may be proepileptogenic, providing a foundation for future research.

The recent study by Kushner et al. (2025) expands on this foundation by providing a systematic electrophysiological characterization of L2/3 PN within the context of childhood epilepsy. The study's unique approach lies in its direct examination of L2/3 PN in the postoperative pediatric tissue from a variety of epilepsies and comparison with the control tissue. The control tissue in this study consists of the cortical tissue obtained during access surgery for subcortical tumors in patients with no history of seizures. Whole-cell patch–clamp recordings were performed on a total of 108 cells following stereotyped protocols. The electrophysiological characteristics of the recorded cells were meticulously evaluated. Kushner and colleagues demonstrated that human L2/3 PNs exhibit heterogeneous electrophysiological profiles. They determined a taxonomy of the neurons through a comprehensive observational process. Neurons were classified into five distinct categories based on their firing behavior: accommodating, regular spiking, notch, stuttering, and early spiking. This classification contrasts with the two subtypes identified by Deitcher et al. and with the well-known homogeneity of L2/3 PNs reported in the rodent cortex. These findings suggest that L2/3 PNs in the epileptic tissue may contribute to increased cell heterogeneity.

Furthermore, the study reveals alterations in the membrane properties of neurons when comparing the epileptic tissue to the control tissue. First, a reduction in sag current amplitude was observed, which suggests a decrease in functional HCN channels located at the membrane. In MCD samples, increased input resistance could favor depolarization, thereby promoting epileptiform activity. Examining the action potential kinetics yielded somewhat counterintuitive results. L2/3 PNs from the epileptic tissue were hypoexcitable, as evidenced by a higher threshold and reduced maximum firing rate, compared with pseudocontrols.

A comparison of MCD samples with other epilepsies revealed significant differences in action potential characteristics, including half-width, amplitude, and firing rate accommodation. This observation emphasizes the potential for differential impairment of epileptic networks, contingent on the specific type of epilepsy and the underlying epileptogenic process.

Finally, the researchers conducted a comprehensive evaluation of the specific effects of 4AP on these tissues. As predicted, 4AP increased the half-width and decreased the threshold of action potentials. These changes are expected to increase calcium influx during action potentials, promoting synaptic transmission and resulting in network hyperexcitability. However, the input–output relationship did not demonstrate any alterations in individual cell excitability. Using 4AP was advantageous because it functions independently of a single neurotransmitter and modulates network excitability. This makes it a suitable tool for studying epilepsy, a network pathology.

Amidst extensive debate about the precise involvement of L2/3 PNs in epileptic activity, Kushner et al. describe the specific electrophysiological and morphological properties of L2/3 PNs in the epileptic and control postoperative pediatric tissue. They provide a nuanced picture, noting characteristics of both hypo- and hyperexcitability. For instance, they include higher input resistance and a reduced maximum firing rate. Furthermore, neurons from the epileptic postoperative tissue exhibited a diminished sag current. Additionally, the authors emphasize the heterogeneity of the recorded L2/3 PNs.

Furthermore, the results indicated significant variations in the action potential kinetics among different types of epilepsy. This finding suggests the presence of mechanisms that are specific to MCD compared with other types of epilepsy. MCD has been linked to somatic mutations that occur during corticogenesis. Hundreds of genes have been identified as contributing factors to the various subtypes of MCD. Many of these genes are involved in the processes of cell–cycle regulation, cytoarchitecture, and cell migration (Guerrini and Dobyns, 2014). However, a correlation between genetic data and functional studies still needs to be established.

The authors provide an indispensable and precise characterization of the effects of 4AP on human pyramidal neurons. 4AP has been used for decades as a model of acute epilepsy. The recent study by Kushner et al. offers significant insights into the epileptogenic effects of 4AP, demonstrating that this compound enhances network excitability with minimal effect on cell excitability by blocking A-type potassium channels. Future studies should be conducted to investigate the potential benefits of opening these channels, such as decreased network excitability and, perhaps, the emergence of an antiepileptic effect.
